# Supplementary material for: Characterization of Two Unique Cold-Active Lipases Derived from a Novel Deep-Sea Cold Seep Bacterium
Source: Microorganisms. 2021 Apr 10;9(4):802. doi: 10.3390/microorganisms9040802 (PMC8069351; doi:10.3390/microorganisms9040802)
Supplement: Supplementary file 1 [file microorganisms-09-00802-s001.pdf]

Supplemental information

Characterization of Two Unique Cold-Active Lipases Derived from a Novel Deep-Sea Cold Seep Bacterium

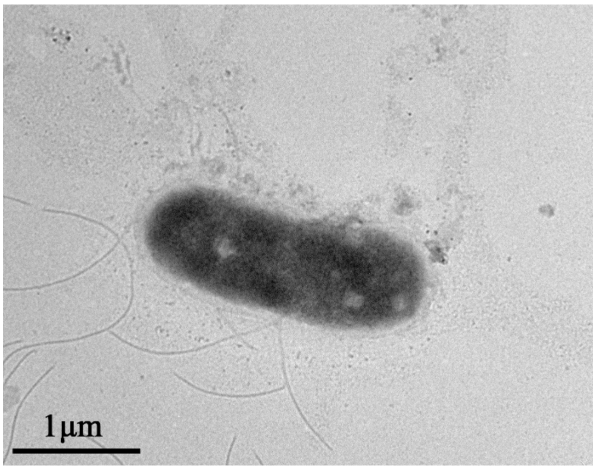

Figure S1. Morphology observation of strain gcc21 by TEM. Bars, 1 μm.

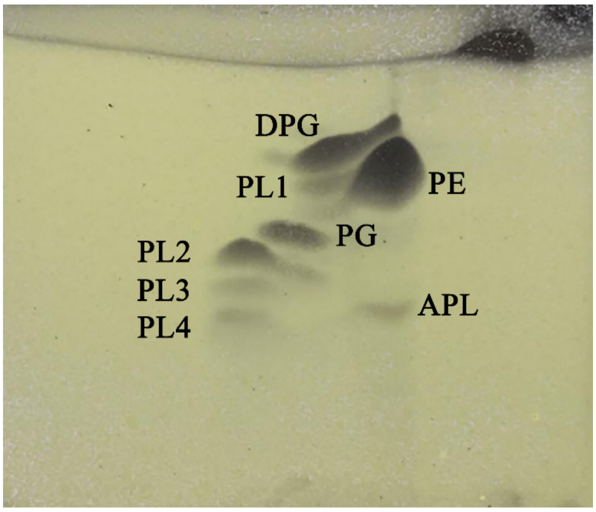

Figure S2. The polar lipids of strain gcc21 as revealed by two-dimensional TLC. Chloroform/methanol/water (65:25:4, vol/vol/vol) was used in the first direction, followed by chloroform/glacial acetic acid/methanol/water (80:18:12:5, vol/vol/vol/vol) in the second direction. The plate was sprayed with 10% ethanolic molybdophosphoric acid. DPG, diphosphatidylglycerol; PG, phosphatidylglycerol; PE, phosphatidylethanolamine; PL 1-4, unidentified phospholipid; APL, unidentified aminophospholipid.

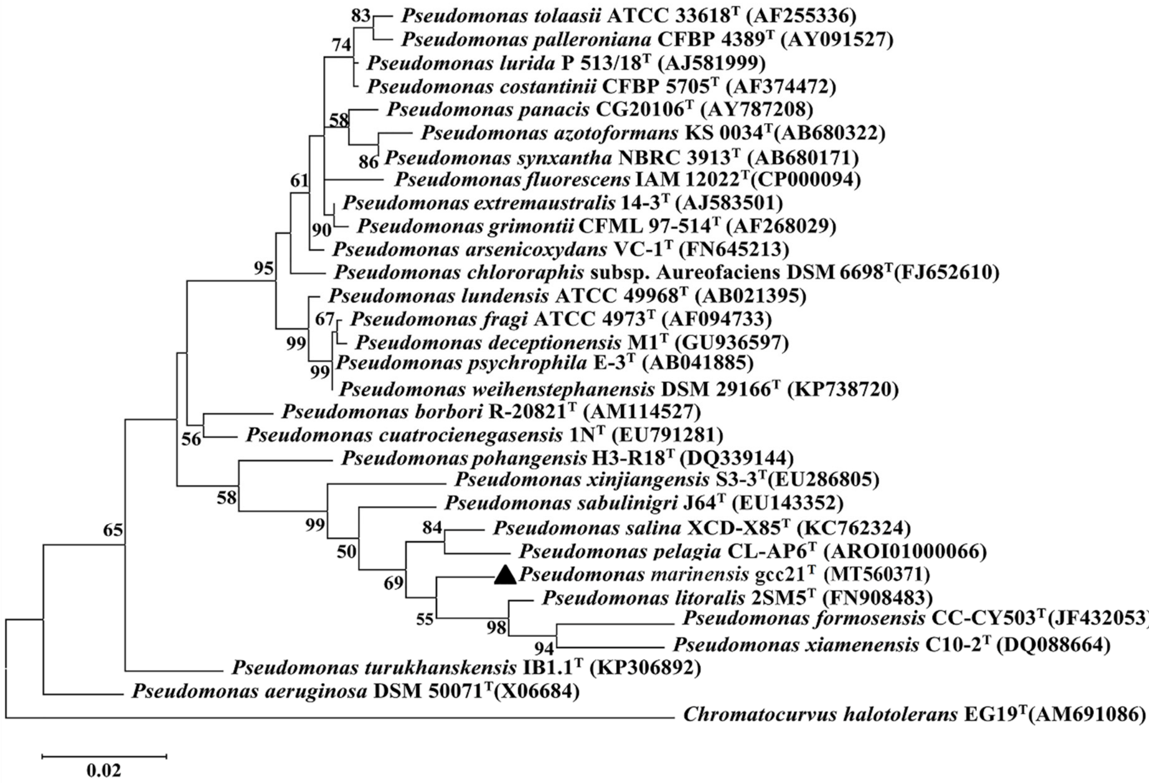

Figure S3. Maximum-likelihood tree based on 16S rRNA gene sequences of strain gcc21^T and related taxa. The sequence of *Chromatocurvus halotolerans* EG19^T was used as an outgroup. Bootstrap values at nodes were derived from 1000 replicates. Only bootstrap values higher than 50% are shown. Bar, 0.02 substitutions per nucleotide position.

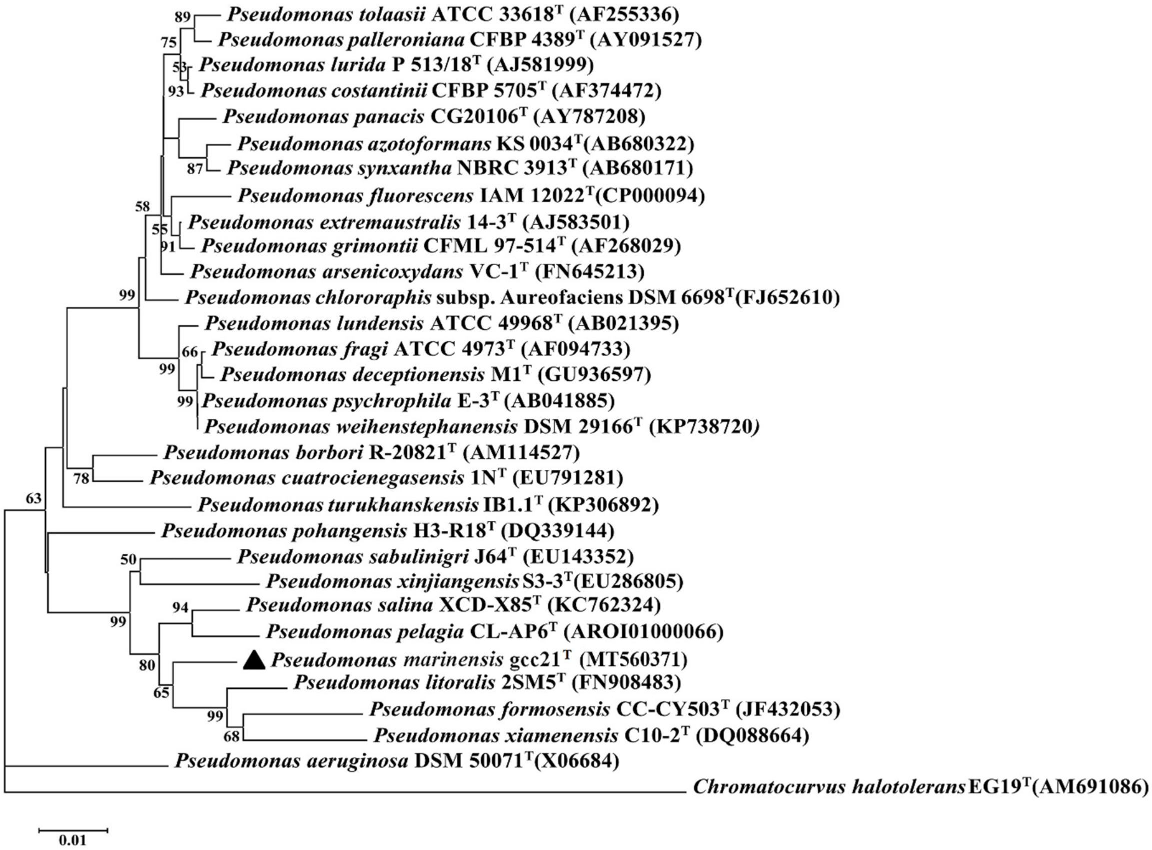

Figure S4. Minimum-evolution tree based on 16S rRNA gene sequences of strain gcc21^T and related taxa. The sequence of *Chromatocurvus halotolerans* EG19^T was used as an outgroup. Bootstrap values at nodes were derived from 1000 replicates. Only bootstrap values higher than 50% are shown. Bar, 0.01 substitutions per nucleotide position.

Table S1. Comparison of the main fatty acids (%) of strain gcc21^T and the type strain *P. aeruginosa* DSM50071^T.

Fatty acid	Percentage (w/v) of total fatty acids	
	**1**	**2**
Branched-:		
$C_{16:0}$	17.38	31.61
$C_{17:0}$ cyclo	16.26	9.13
Summed features:*		
3	15.99	4.77
8	26.87	18.97

Strains: 1, gcc21^T (all data from this study); 2, *Pseudomonas. aeruginosa* DSM50071^T (all data from this study).

Table S2. ANIb, ANIm and *is*DDH values between the genome sequence of strain gcc21^T and closely related *Pseudomonas* species.

Strains	Accession no.	ANIb value (%)	ANIm value (%)	*is*DDH value (%)
1	LT629763	73.00	83.83	19.70
2	CP012001	70.25	83.23	19.50
3	KC762324	72.83	84.14	19.60
4	FN908483	74.19	84.30	20.40
5	ARIO01000066	72.86	83.96	19.90

Strains: 1, *Pseudomonas sabulinigri* J64^T (all data from this study); 2, *Pseudomonas aeruginosa* DSM50071^T (all data from this study); 3, *Pseudomonas salina* XCD-X85^T (all data from this study); 4, *Pseudomonas litoralis* 2SM5^T (all data from this study); 5, *Pseudomonas pelagia* CL-AP6^T (all data from this study). ANI: Average Nucleotide Identity; ANIb: ANI based on the BLASTN algorithm; ANIm: ANI based on the MUMMER ultra-rapid aligning tool; isDDH: the in silico DNA-DNA similarity values.

Table S3. Amino acid composition of Lipase 1, Lipase 2 and others lipases.

Lipase	Amino acid composition (%)					
	**Gly (G)**	**Cys (C)**	**Ser (S)**	**Phe (F)**	**Asn (N)**	**Tyr (Y)**
1	15.8	1.4	7.3	3.8	5.2	2.4
2	15	1.3	10.2	4.4	7.1	3.4
3	14.4	-	8.6	4.6	5.4	3.7
4	13.9	-	8.4	4.0	4.8	4.2
5	13.8	-	7.9	4.0	4.8	4.2
6	8.8	0.3	7.5	4.0	5.8	2.7

Source of lipases: 1, Lipase 1; 2, Lipase 2; 3, *Pseudomonas* sp. AMS8 (ADM87309); 4, *Pseudomonas* sp. KB700A (BAB64913); 5, *Pseudomonas* sp. TK-3 (BAM05474)); 6, *Pseudomonas fragi* (WP 016781240).
